# Stage-Specific Inhibition of TrkB Activity Leads to Long-Lasting and Sexually Dimorphic Effects on Body Weight and Hypothalamic Gene Expression

**DOI:** 10.1371/journal.pone.0080781

**Published:** 2013-11-29

**Authors:** Mardi S. Byerly, Roy D. Swanson, G. William Wong, Seth Blackshaw

**Affiliations:** 1 Department of Physiology, Johns Hopkins University School of Medicine, Baltimore, Maryland, United States of America; 2 Center for Metabolism and Obesity Research, Johns Hopkins University School of Medicine, Baltimore, Maryland, United States of America; 3 Department of Neuroscience, Johns Hopkins University School of Medicine, Baltimore, Maryland, United States of America; 4 Center for High throughput Technology, Johns Hopkins University School of Medicine, Baltimore, Maryland, United States of America; University of Minnesota, United States of America

## Abstract

During development, prenatal and postnatal factors program homeostatic set points to regulate food intake and body weight in the adult. Combinations of genetic and environmental factors contribute to the development of neural circuitry that regulates whole-body energy homeostasis. Brain-derived neurotrophic factor (Bdnf) and its receptor, Tyrosine kinase receptor B (TrkB), are strong candidates for mediating the reshaping of hypothalamic neural circuitry, given their well-characterized role in the central regulation of feeding and body weight. Here, we employ a chemical-genetic approach using the *TrkB^F616A/F616A^* knock-in mouse model to define the critical developmental period in which TrkB inhibition contributes to increased adult fat mass. Surprisingly, transient TrkB inhibition in embryos, preweaning pups, and adults all resulted in long-lasting increases in body weight and fat content. Moreover, sex-specific differences in the effects of TrkB inhibition on both body weight and hypothalamic gene expression were observed at multiple developmental stages. Our results highlight both the importance of the Bdnf/TrkB pathway in maintaining normal body weight throughout life and the role of sex-specific differences in the organization of hypothalamic neural circuitry that regulates body weight.

## Introduction

Neuropeptides and other secreted proteins expressed in the hypothalamus play a critical role in modulating body weight and food intake in adult animals [1], [2]. Hypothalamic energy balance is modulated by first-order neurons of the hypothalamus. The hypothalamus contains two populations of neurons—orexigenic [expressing agouti related protein (Agrp), and neuropeptide Y (Npy)] and anorexigenic [expressing pro-opiomelanocortin (Pomc) and cocaine- and amphetamine-regulated transcript (Cart)] neurons—that make up the central melanocortinergic system to modulate energy balance [3], [4]. Many of the same factors also actively shape the embryonic and early postnatal development of the hypothalamic neural circuitry [1], [2], [5], [6]. Dietary cues act in a critical period during prenatal and early postnatal development to regulate homeostatic set points that modulate food intake and body weight in the adult, a process known as metabolic imprinting [2]. The effects of metabolic imprinting are sexually dimorphic. Male rats that are undernourished *in utero* have reduced body weight as young adults, while females exhibit increased body weight [7].

The neurotrophic factor Bdnf and its receptor, TrkB, play critical roles in the development of neural circuitry that modulates food intake and body weight. Their expression levels are modulated by genetic and dietary factors [8]–[11]. A missense mutation in human *TRKB* (*NTRK2*) results in severely obese children [12]; similarly, deletion of *Bdnf* results in obese mice [13]–[18]. However, the precise timing when TrkB signaling induces obesity during pre- and postnatal development is unknown. We addressed this question using a chemical-genetic approach whereby TrkB signaling can be chemically inhibited in a spatiotemporal and reversible manner in the *TrkB^F616A/F616A^* knock-in mouse model [19], [20].

The different components of hypothalamic circuitry that control feeding mature at different stages during development. The vast majority of hypothalamic neurogenesis occurs between embryonic (E) day 10 and E16 in mice [21]. During embryonic development, *TrkB* expression is restricted to the CNS and the cranial and dorsal root ganglia [22]. Within the hypothalamus, *TrkB* is broadly expressed in regions that regulate food intake [paraventricular nucleus (PVN), dorsomedial hypothalamus (DMH), ventromedial hypothalamus (VMH), arcuate nucleus (ARC), and lateral hypothalamus (LH)].

The axonal connections of different hypothalamic neuronal subtypes mature at different rates. Some projections, such as those from the VMH to the PVN, appear to be fully developed by birth. On the other hand, projections from the ARC to the PVN or the ARC to the LH do not fully develop until the end of the second postnatal week [1], [23], [24]. Moreover, robust *Bdnf* expression persists in the VMH and other hypothalamic nuclei into adulthood [25]. Thus, there is a broad range of developmental stages at which altered Bdnf/TrkB signaling could lead to lasting changes in hypothalamic neuronal connectivity. In this study, we used a chemical-genetic approach to delineate when TrkB signaling is required during development to establish hypothalamic neural circuitry that is critical for the proper maintenance of adult energy balance.

## Materials and Methods

### Animals and 1NMPP1 inhibition of TrkB

**Figure 1:**
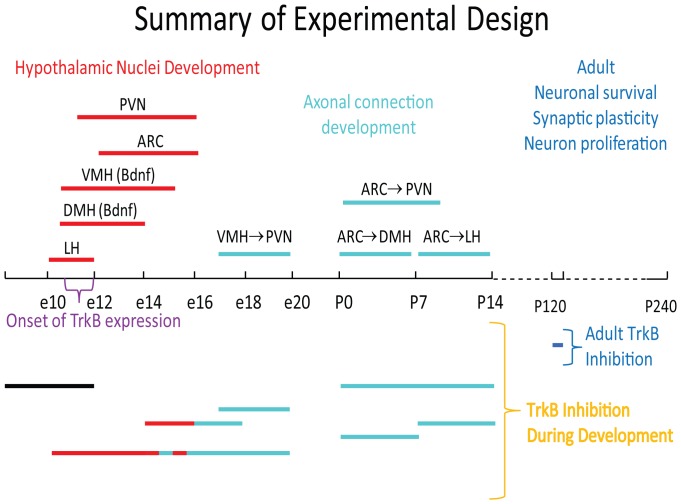
Summary of experimental design of pharmacological TrkB inhibition by 1NMPP1. 1NMPP1 was delivered to pre- or postnatal pups at the specified times. Bars (bottom) represent the time period for 4 embryonic treatments, 3 postnatal treatments, and one treatment given during adulthood. Developmental events occurring during the various treatment periods are indicated in red (embryonic), cyan (post-natal), or blue (adult). PVN = paraventricular nucleus; ARC = arcuate nucleus; VMH (Bdnf) = ventromedial hypothalamus (brain-derived neurotrophic factor); DMH (Bdnf) = dorsomedial hypothalamus (brain-derived neurotrophic factor); LH = lateral hypothalamus.

*TrkB^F616A/F616A^* mice were used [20], [26] and obtained from Dr. David Ginty at Johns Hopkins University School of Medicine (Baltimore, MD). Mice were initially screened at many different stages, with multiple litters generated for in-depth investigation of sex-specific effects for groups of interest (control, 5 litters; E0–E12, 2 litters; E17–E20, 2 litters; E8–E20, 4 litters). A point mutation was introduced into *TrkB* to convert phenylalanine to alanine at position 616 (F616A) through targeted gene replacement, which allows pharmacological and temporal inhibition of TrkB signaling via the highly membrane-permeable small molecule 1NMPP1 [20]. 1NMPP1 was provided in the drinking water of the dam, since it can readily cross the placenta or be secreted via the dam's milk, be ingested and cross the blood-brain barrier [27] during a temporally specified pre- or postnatal developmental window. Vehicle with no 1NMPP1 was provided to control animals. 1NMPP1 delivery to the pregnant mother inhibits TrkB receptor during embryonic and postnatal development [20]. The treatment group received 1NMPP1 (80 µM) either during embryonic development via the mother's drinking water for the specified duration (Figure 1) or as an adult mouse for 7 days via drinking water beginning at 4 months of age. Fresh water was made every two days to ensure chemical integrity, although 1NMPP1 is stable for at least three days in room temperature water [20]. 1NMPP1 was removed after the designated treatment duration to allow TrkB signaling reactivation [20], which has been shown to occur two hours after removal of the 1NMPP1 [20]. All animals were provided *ad libitum* access to standard laboratory chow (2018 Teklad, Harlan Laboratories) and water while maintained on a 12∶12 hour light-dark cycle in a vivarium for rodent housing with controlled temperature and humidity. All studies were conducted in accordance with the recommendations provided by the *Guide for the Care and Use of Laboratory Animals* of the National Institutes of Health and approved by the Animal Care and Use Committee of The Johns Hopkins University School of Medicine (protocol numbers MO10M108 and MO11M49).

### Body weight and body composition measurements

Body weight changes were monitored after 1NMPP1 treatment in postnatal mice as early as 6 days of age until as late as 4 months of age, and until 8 months of age in the adult mouse. Fat mass, fat-free mass (i.e., lean mass), and water content were assessed to determine body composition using a whole-body NMR machine (EchoMRI, Waco, TX) as previously described [28], [29]. Fat mass, fat-free mass, and water content values were generated after animals were placed into an immobilizing tube and scanned twice. The average of the two scans is presented.

### RNA extraction and quantitative real-time PCR (qPCR)

Mouse hypothalami were dissected in sagittal planes utilizing anatomical landmarks (e.g., the anterior commissure and the oculomotor nerve). RNA was extracted using RNeasy Midi kits (Qiagen, Valencia, CA) and quantified using a Nanodrop 1000 spectrophotometer (Thermo Scientific, Waltham, MA). Superscript II reverse transcriptase (Life Technologies, Carlsbad, CA) and random primers (Life Technologies, Carlsbad, CA) were used to generate cDNA from 1 µg of RNA. Applied Biosystems (Life Technologies, Carlsbad, CA) quantitative real-time PCR SYBR green master mix was used to quantify mRNA levels. ΔC_t_ values were generated by normalizing the sample C_t_ value to the C_t_ value for *18S rRNA*. Samples were run in duplicate for each primer and the primers for each target were located in separate wells. The 2^−ΔΔCt^ value (relative mRNA levels) was then generated by normalizing the data to control (non-treated) animals [30]. The following primer pairs were used: *Agrp* forward – 5′ ATGCTGACTGCAATGTTGCTG 3′; *Agrp* reverse – 5′ CAGACTTAGACCTGGGAACTCT 3′; *Mc4r* forward – 5′ CGGACGGAGGATGCTATGAG 3′; *Mc4r* reverse – 5′ CGCCACGATCACTAGAATGTT 3′; *Pomc* forward – 5′ ATGCCGAGATTCTGCTACAG 3′; *Pomc* reverse – 5′ TGCTGCTGTTCCTGGGGC 3′; *Npy* forward – 5′ ATGCTAGGTAACAAGCGAATGG 3′; *Npy* reverse – 5′ TGTCGCAGAGCGGAGTAGTAT 3′; Leptin receptor (*LepR*) forward – 5′ TGGTCCCAGCAGCTATGGT 3′; *LepR* reverse – 5′ ACCCAGAGAAGTTAGCACTGT 3′; *18S rRNA* forward – 5′ GCAATTATTCCCCATGAACG 3′; and *18S rRNA* reverse- 5′ GGCCTCACTAAACCATCCAA 3′.

### *In situ* hybridization (ISH)

Brains were harvested for histological analysis at P90, fresh frozen on dry ice in OCT compound, and then stored at −80°C. *Pomc* and *Agrp* cDNA were used as templates to generate riboprobes using the T3 or T7 RNA polymerase (Roche) for 2 hours at 37°C. Riboprobes were precipitated with LiCl. Non-radioactive ISH was performed as previously described [31], [32], with the following minor modifications: slides were incubated twice in 0.2x SSC (3 M NaCl and 300 mM sodium citrate) at 65°C for 30 minutes, washed in 0.2x SSC for 5 minutes, then washed with 0.1 M Tris pH 7.5, 0.15 M NaCl for 5 minutes, and blocked with 2 mL 0.1 M Tris pH 7.5, 0.15 M NaCl plus 0.1% HISS (Heat Inactive Sheep Serum, S2263, Sigma-Aldrich, St. Louis, MO, USA) for 1 hr. ImageJ (http://rsbweb.nih.gov/ij/) was used to perform semi-quantitative analysis of gene expression.

### Statistical analysis

Statistical analyses were conducted using a one-way ANOVA to identify individual differences between groups or repeated-measures ANOVA with a Fisher LSD post-hoc analysis to identify differences between groups over days (Statistica, v.8.0, Tulsa, OK). *P*<0.05 was considered significant, and values are reported as means ± SEM.

## Results

### Reversible TrkB inhibition during pre- and postnatal stages of hypothalamic development

To determine the critical time period at which TrkB signaling regulates development of body weight and/or body composition during pre- and postnatal development, we used the point mutant knock-in mouse model (*TrkB^F616A/F616A^*) that allows specific and reversible inhibition of TrkB function via the small molecule 1NMPP1 [20]. 1NMPP1 was administered for fixed intervals during pre- and postnatal development and in adults. Removal of 1NMPP1 from the drinking water allowed reactivation of TrkB signaling. The small molecule 1NMPP1 was initially developed to target the enzyme activity of protein kinases, in combination with genetic manipulation [19], [33]. *In vitro*, TrkB phosphorylation is restored 2 hours after removal of 1NMPP1 [20]. *In vivo*, 1NMPP1 is able to penetrate the brain, and is rapidly metabolized, allowing reactivation of the receptor by 48 hours after removal of 1NMPP1 from the drinking water [27]. Since TrkB is primarily expressed in the central and peripheral nervous system during development [22], [34], [35], we inhibited TrkB signaling during known periods of hypothalamic nuclei formation, as well as during formation of axonal connections between hypothalamic nuclei known to regulate body weight (see Figure 1).

Hypothalamic neurons organize into many different nuclei during development, ranging from as few as 12 distinct nuclei to more than 28 [36]. The formation of hypothalamic nuclei begins at E10 and continues until E16, with the bulk of neurogenesis occurring from E12–14 [21], [37], [38]. The most lateral hypothalamic nuclei are generated first, followed by the more medial nuclei. The LH begins developing on E10, followed by DMH, VMH and PVN on E10.5 and ARC on E11 [21], [37], [39]. Axonal connections between nuclei form during both pre- and postnatal periods: axonal projections from the VMH reach the PVN by E17.5; ARC projects to DMH by postnatal (P) 6; efferent projections from the ARC reach the PVN by P8-P10; while ARC efferent projections reach the LH at P12 [1], [24], [40]. Knowing this, we initially tested the effects of TrkB inhibition at intervals corresponding to specific events in hypothalamic development (Figure 1): hypothalamic neurogenesis and nucleogenesis from E14-E18; formation of VMH to PVN projections from E17–E20; hypothalamic neurogenesis, nucleogenesis and early stages of synaptogenesis from E8–E20; formation of both ARC to DMH and ARC to PVN projections from P0–P6; formation of ARC to LH projections from P7–P14; formation of most major intrahypothalamic connections from P0–P14; and E0–E12, which was prior to or at the onset of TrkB expression (E10.5–E12: [22], [41]), as an additional control group.

**Figure 2:**
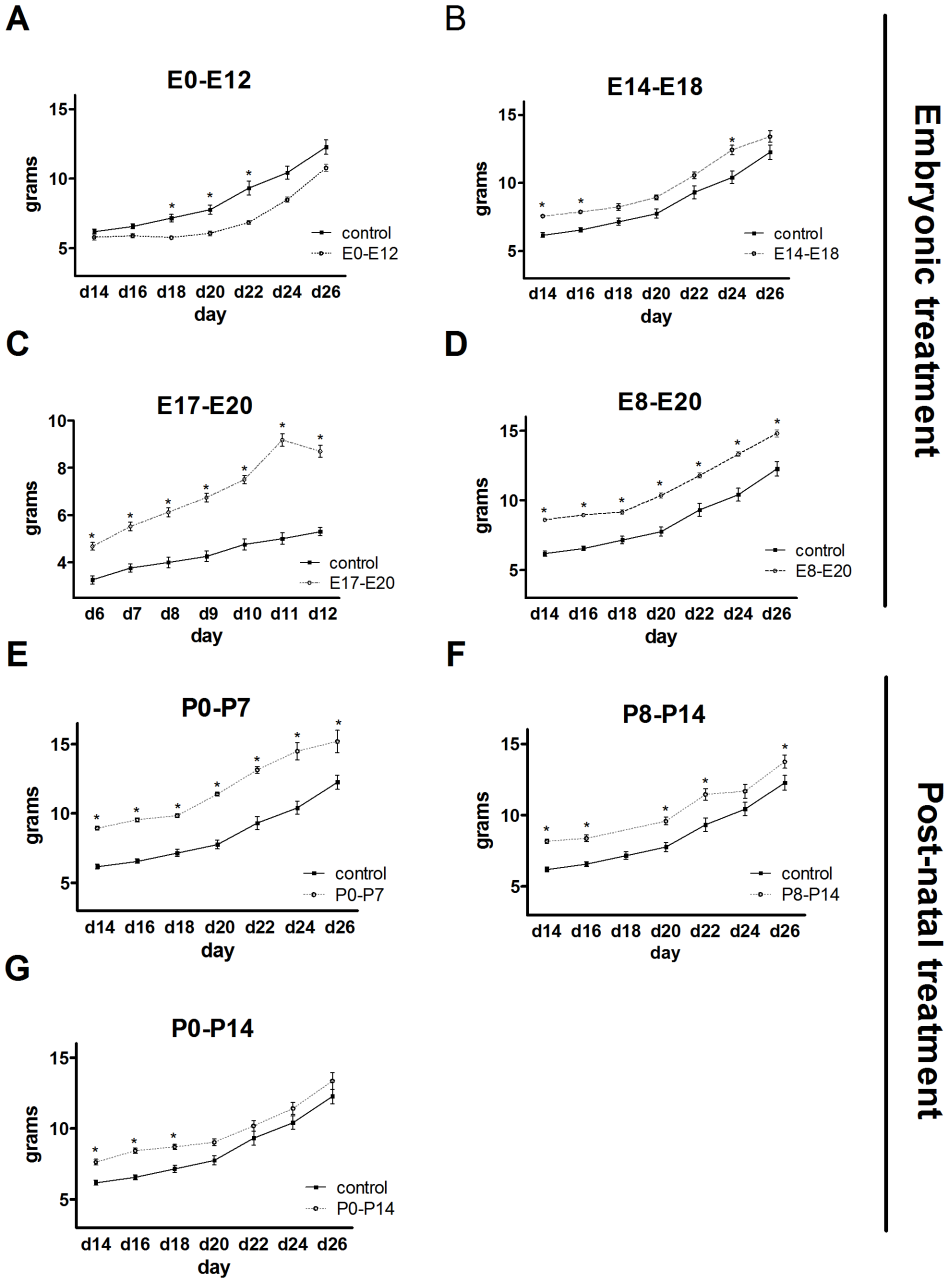
Screen for stage-specific effects of TrkB inhibition and postnatal changes in body weight. Body weight measurements of mice treated with 1NMPP1 A) prior to or at the onset of TrkB gene expression (E0–E12), B) during the formation of hypothalamic nuclei (E14–E18), C) during the time period that projections form between nuclei of the VMH (E17–E20), D) during all embryonic development (E8–E20), E) during formation of connections between the ARC and DMH or PVN (P0–P7), F) during the formation of ARC connections between the ARC and LH (P8–P14), and G) during formation of all nuclei connections (P0–P14). Data presented in grams at the indicated days. Open circles denote 1NMPP1 treatment, while closed squares denote controls (vehicle only). Data shown are mean ± SEM for each group. *Significant relative to control, *P*<0.05.

Early postnatal stages of body weight were screened and measured after 1NMPP1 treatment at different embryonic phases (control treatment versus 1NMPP1, Figure 2). The delivery of 1NMPP1 prior to or at the onset of TrkB expression (E0–E12) transiently and modestly decreased body weight (Figure 2A) (E0–E12, n = 5; control, n = 22, *F*_1,27_ = 58.57; Day 14, *P* = 0.60; Day 16, *P* = 0.38; Day 18, *P* = 0.07; Day 20, *P* = 0.03; Day 22, *P* = 0.002; Day 24, *P* = 0.01; Day 26, *P* = 0.06). Likewise, TrkB inhibition during formation of hypothalamic nuclei (E14–E18) transiently and modestly increased body weight (Figure 2B) (E14–E18, n = 7; vs. control, n = 22, *F*_1,29_ = 66.82; Day 14, *P* = 0.03; Day 16, *P* = 0.04; Day 18, *P* = 0.10; Day 20, *P* = 0.07; Day 22, *P* = 0.06; Day 24, *P* = 0.002; Day 26, *P* = 0.08). On the other hand, TrkB inhibition during E17–E20 induced a stable increase in body weight over all days, which resulted in a robust 45–91% increase in body weight relative to control animals that was not transient (Figure 2C) (E17–E20, n = 5; control, n = 22, *F*_1,14_ = 40.83; Day 6, *P* = 0.00002; Day 7, *P* = 10^−6^; Day 8, *P* = 10^−6^; Day 9, *P* = 10^−6^; Day 10, *P* = 10^−6^; Day 11, *P* = 10^−6^; Day 12, *P* = 10^−6^). TrkB inhibition during E8-E20 less robustly increased body weight, 27% relative to controls (Figure 2D) (E8–E20, n = 18; control, n = 22, *F*_1,40_ = 77.69; Day 14, *P* = 0.00005; Day 16, *P* = 8×10^−6^; Day 18, *P* = 0.0001; Day 20, *P* = 6×10^−6^; Day 22, *P* = 0.00005; Day 24, *P* = 3×10^−6^; Day 26, *P* = 7×10^−6^). These data suggest that TrkB inhibition in the CNS during acute developmental time windows affects body weight and body composition. Therefore, we analyzed phenotype differences between the E17–E20 and E8–E20 groups in more detail; those results are presented later.

Acute postnatal TrkB inhibition increased body weight when the ARC develops synaptic projections to the DMH (P0–P7), PVN (P0–P8) or LH (P8–P10). TrkB inhibition from P0–P7, P8–P14, and P0–P14 increased body weight by 49%, 102%, and 49%, respectively, relative to controls (Figure 2E–G, respectively) (P0–P7, n = 4; control, n = 22, *F*_1,26_ = 57.49; Day 14, *P* = 0.002; Day 16, *P* = 0.001; Day 18, *P* = 0.002; Day 20, *P* = 0.00009; Day 22, *P* = 0.00004; Day 24, *P* = 0.00001; Day 26, *P* = 0.001) (P8–P14, n = 7; control, n = 22, *F*_1,28_ = 62.89; Day 14, *P* = 0.005; Day 16, *P* = 0.01; Day 20, *P* = 0.01; Day 22, *P* = 0.003; Day 24, *P* = 0.07; Day 26, *P* = 0.03) (P0–P14, n = 7; control, n = 22, *F*_1,29_ = 62.12; Day 14, *P* = 0.04; Day 16, *P* = 0.01; Day 18, *P* = 0.03; Day 20, *P* = 0.07; Day 22, *P* = 0.22; Day 24, *P* = 0.16; Day 26, *P* = 0.13). This suggests that TrkB signaling during the formation of synaptic projections from the ARC to the LH neurons may play a critical role in modulating body weight.

### TrkB inhibition during pre- and postnatal development induces long-lasting alterations in body weight and body composition

**Figure 3:**
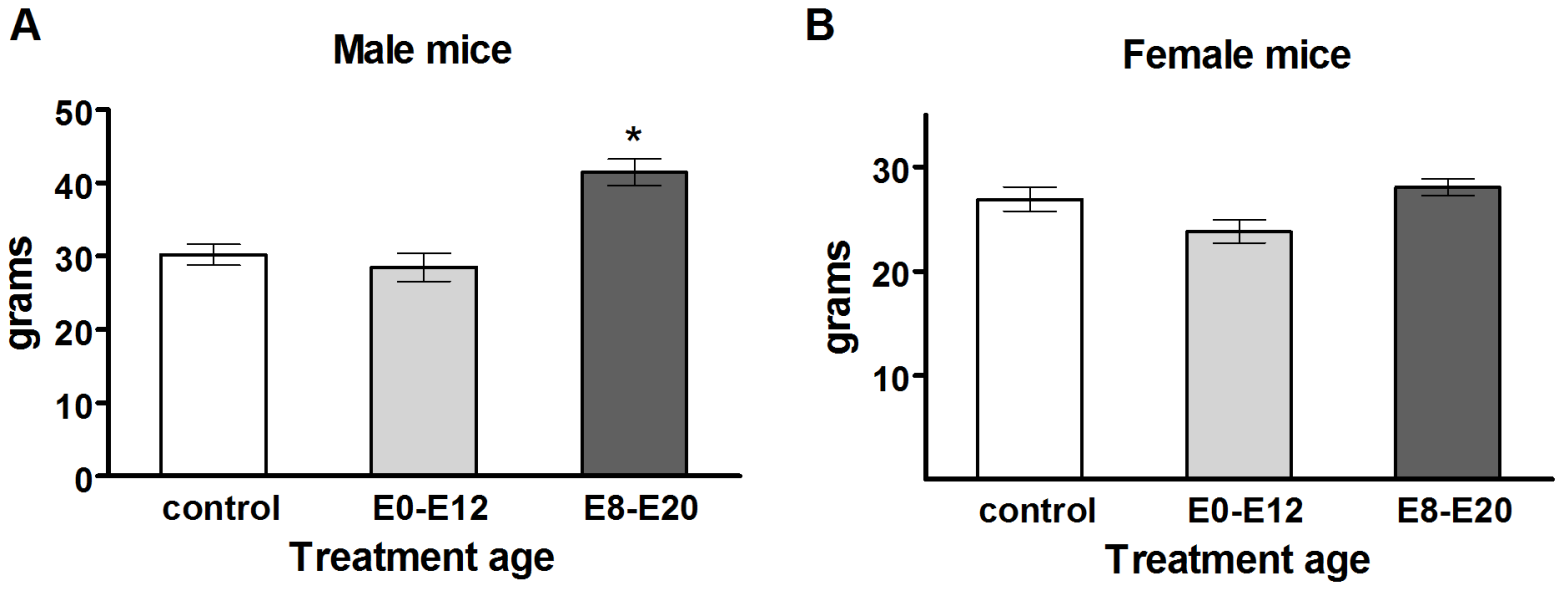
Long-term body weight changes after acute prenatal TrkB inhibition. Four months after 1NMPP1 treatment, body weight of A) male or B) female mice. For each, control mice are compared with those treated at E0–E12 or E8–E20. Data shown are presented in grams, mean ± SEM for each group. *Significant relative to control, *P*<0.05.

We observed both transient and long-lasting effects on body weight resulting from chemical inhibition of TrkB, the magnitude of which depends on the timing of treatment during development. TrkB inhibition between E8 and E20 dramatically increased body weight over the first three postnatal weeks. Long-term changes in body weight were still observed in both male and female mice at four months of age, with increased body weight in groups treated with 1NMPP1 at E8–E20, but not at E0–E12, relative to controls. The reduction in the size of this initial spike of postnatal body weight over time may be related to establishment of the leptin feedback system during postnatal development [42], [43]. Furthermore, male mice showed long-term increases in body weight after 1NMPP1 treatment at E8–E20 (Figure 3A, male mice) (control, n = 10, 30.1 g±1.6; E0–E12, n = 6, 28.5 g±1.9; E8–E20, n = 9, 41.4 g±1.8) (control vs. E8–E20, *F*_1,19_ = 31.64, *P* = 0.00003; control vs. E0–E12, *F*_1,16_ = 0.65, *P* = 0.43), whereas female mice did not have increased body weight after 1NMPP1 treatment at E8–E20 (Figure 3B, female mice) (control, n = 12, 26.9 g±1.16; E0–E12, n = 6, 23.8 g±1.13; E8–E20, n = 12, 28.1 g ± 0.8) (control vs. E8–E20, *F*_1,23_ = 3.09, *P* = 0.09; control vs. E0–E12, *F*_1,17_ = 0.51, *P* = 0.48). TrkB inhibition from E0–E12 did not alter body weight over the long-term in male or female mice, relative to control mice (Figure 3A-B, respectively). This suggests TrkB inhibition leads to sex-specific effects on body weight regulation and that these effects may depend on the developmental time window of TrkB inhibition.

**Figure 4:**
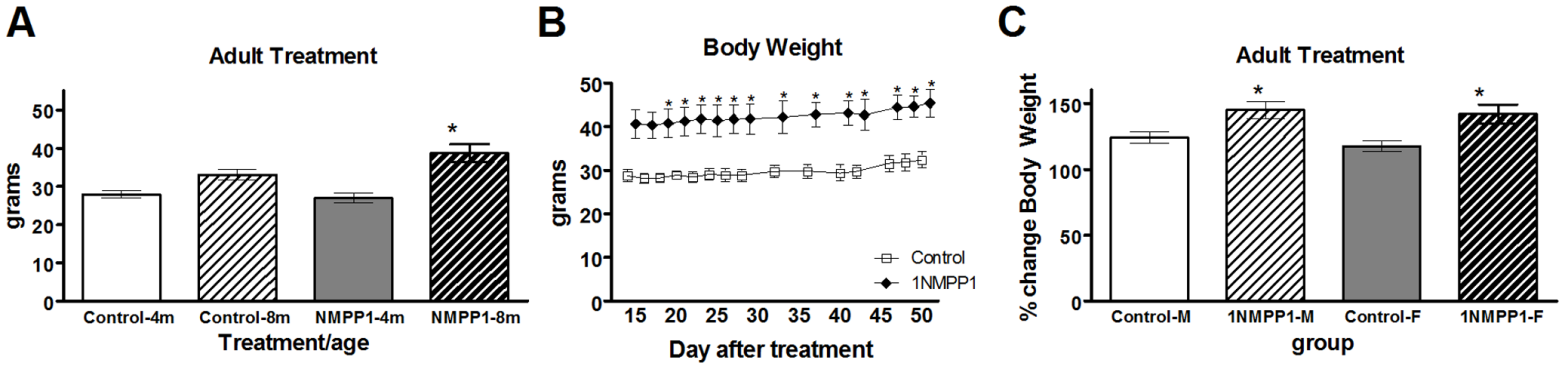
Long-term body weight changes after acute TrkB inhibition in adult female mice. A) Body weight measurements of adult control mice or mice treated with 1NMPP1 at four months of age (4 m) and eight months of age (8 m), in grams. Mice were treated with 1NMPP1 for 7 days, followed by no 1NMPP1 for the remaining duration. B) Body weight measurements of mice 14–50 days after 1NMPP1 treatment or control, in grams. Open squares denote controls, while closed diamonds denote 1NMPP1-treated mice. C) Percent change in body weight measurements of male (M) or female (F) adult control mice or four months after treatment with 1NMPP1 (8 months of age). Data shown are mean ± SEM for each group. *Significant difference, *P*<0.05.

In adult female mice, a rapid and stable increase in body weight was observed following seven days of TrkB inhibition, in sharp contrast to the phenotype after developmental TrkB inhibition. Prior to TrkB inhibition, body weight was not different between groups (Figure 4A) (control-4m, n = 12, 27.9±0.89; 1NMPP1-4m, n = 10, 27.0±1.3) (*F*_1,22_ = 0.90, *P* = 0.35). Four-month-old animals were treated with 1NMPP1 for seven days and monitored for body weight changes over the subsequent four months. By eight months of age, 1NMPP1-treated mice gained 37% more body weight relative to controls (Fig. 4A) (control-8m, n = 12, 33.0 g±1.5; 1NMPP1-8m, n = 10, 38.6 g±2.3) (*F*_1,22_ = 4.45, *P* = 0.04). The rate of body weight gain after initial TrkB inhibition was rapid and irreversible, as represented by stable body weight differences between groups observed during the first 50 days following vehicle or 1NMPP1 delivery (Fig. 4B) (control-4m, n = 4; 1NMPP1-4m, n = 6, *F*_1,10_ = 6.56; Day 14, *P* = 0.055; Day 16, *P* = 0.050; Day 18, *P* = 0.047; Day 20, *P* = 0.047; Day 22, *P* = 0.035; Day 24, *P* = 0.037; Day 26, *P* = 0.029; Day 28, *P* = 0.026; Day 32, *P* = 0.037; Day 36, *P* = 0.041; Day 40, *P* = 0.031; Day 42, *P* = 0.031; Day 46, *P* = 0.039; Day 48, *P* = 0.039; Day 50, *P* = 0.033). Although TrkB signaling was reactivated after 1NMPP1 removal [20], TrkB failed to restore or reverse the trend in body weight gain over time. We also observed a similar trend of irreversible weight gain after short-term inhibition of TrkB signaling in adult male mice. 1NMPP1 treated male mice gained significantly more weight over four months post-treatment than control mice (45% vs. 24%, control, n = 5; 1NMPP1, n = 4, *F*_1,9_ = 9.34, *P* = 0.018) or female mice (42% vs. 12%, control, n = 6; 1NMPP1, n = 7, *F*_1,13_ = 9.11, *P* = 0.011) (Figure 4C). This suggests that short-term (seven days) TrkB inhibition in adult mice may lead to proportionally more weight gain in females than males.

### Effects of prenatal TrkB inhibition on body composition and hypothalamic gene expression are sexually dimorphic

**Figure 5:**
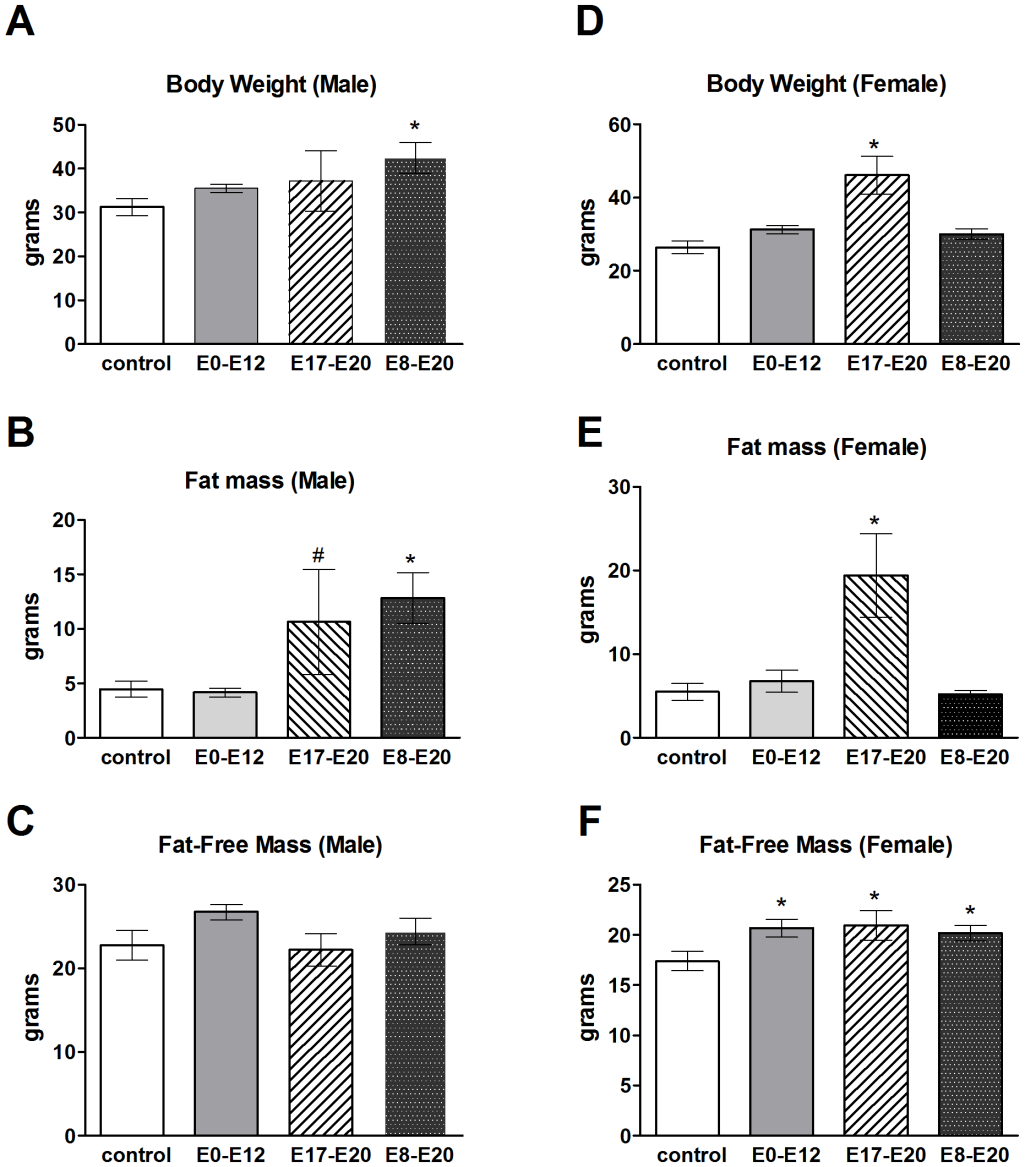
Sex-specific body weight and body composition changes after embryonic TrkB inhibition. A) Body weight, B) fat mass, and C) fat-free mass measurements at four months of age for male control mice or mice receiving 1NMPP1 treatment at E0–E12, E17–E20, or E8–E20. D) Body weight, E) fat mass, and F) fat-free mass measurements at four months of age for female control mice or those receiving 1NMPP1 treatment at E0–E12, E17–E20, or E8–E20. Data shown are mean ± SEM for each group. *Significant difference, *P*<0.05; #*P* = 0.06.

TrkB is primarily expressed in the central nervous system during embryonic development [22], [34], [35]. This allows us to observe the effects of TrkB inhibition during embryonic development on long-term alterations in body weight and hypothalamic gene expression patterns. Interestingly, alterations in body composition and body weight emerged differentially depending on the embryonic period of TrkB inhibition (E17–E20 vs. E8–E20). A striking sexual dimorphism was observed in both body weight and hypothalamic gene expression in the E17–E20 and the E8–E20 treatment groups. Males exhibited significantly increased body weight and fat mass when TrkB was inhibited at E8–E20 (E8–E20, n = 4; control, n = 5: body weight, *F*_1,9_ = 11.73, *P* = 0.009; fat mass, *F*_1,9_ = 63.92, *P* = 0.00009), but not at E17–E20 (Figure 5A and B) (E17–E20, n = 4; control, n = 5: body weight, *F*_1,9_ = 1.76, *P* = 0.22; fat mass, *F*_1,9_ = 5.01, *P* = 0.06). However, when TrkB was inhibited at E17–E20, female mice increased body weight (E17–E20, n = 5; control, n = 7, *F*_1,12_ = 21.39, *P* = 0.0009) and fat mass (E17–E20, n = 5; control, n = 7, *F*_1,12_ = 13.05, *P* = 0.004) (Figure 5D and E). There was no difference in fat-free mass for male mice treated with 1NMPP1 during E17–E20 or E8–E20, but female mice did have increased fat-free mass relative to controls at E17–E20, E8–E20 and when TrkB was inhibited at the onset of expression, E0–E12 (Figure 5C and F, respectively) (female fat-free mass: E17–E20, n = 5; control, n = 7, *F*_1,12_ = 5.36, *P* = 0.043; E8–E20, n = 9; control, n = 7, *F*_1,16_ = 5.94, *P* = 0.028; E0–E12, n = 4; control, n = 7, *F*_1,11_ = 6.24, *P* = 0.033).

**Figure 6:**
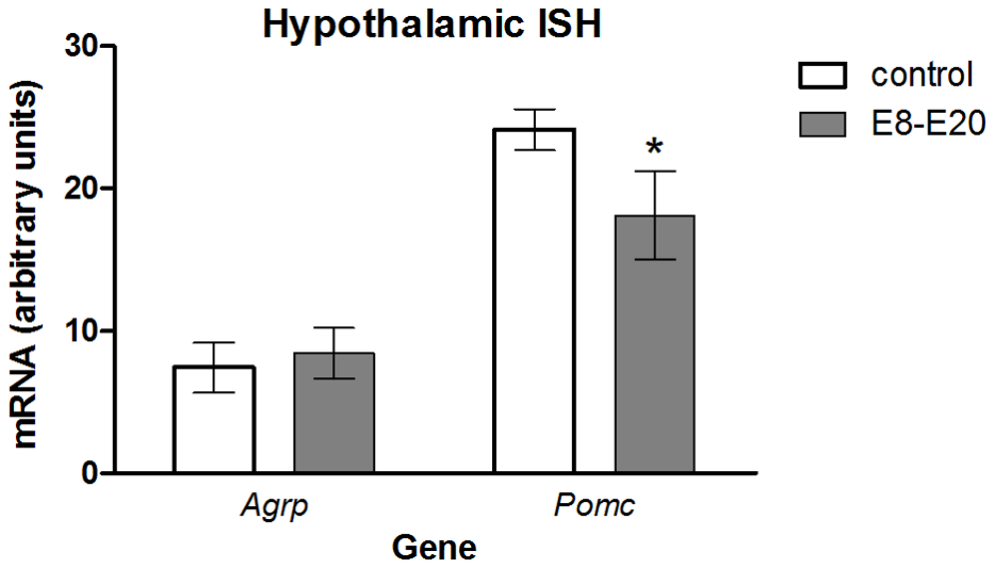
Hypothalamic gene expression levels following stage-specific TrkB inhibition. Brain tissues were harvested from mice at four months of age and stained by *in situ* hybridization (ISH) with probes to *Agrp* and *Pomc*. mRNA levels were quantified for female control mice and after 1NMPP1 treatment at E8–E20. Data shown are mean ± SEM for each group. *Significant difference from all other groups, *P*<0.05.

Next, we determined whether body composition and body weight differences between male and female mice were due to sexually dimorphic expression of orexigenic and anorexigenic neuropeptide genes in the hypothalamus. ISH analysis of female hypothalami revealed decreased *Pomc* mRNA levels in mice treated at E8–E20 (E8–E20, n = 4; control, n = 7, *F*_1,11_ = 5.14, *P* = 0.048) with no change in *Agrp* mRNA levels (Figure 6).

**Figure 7:**
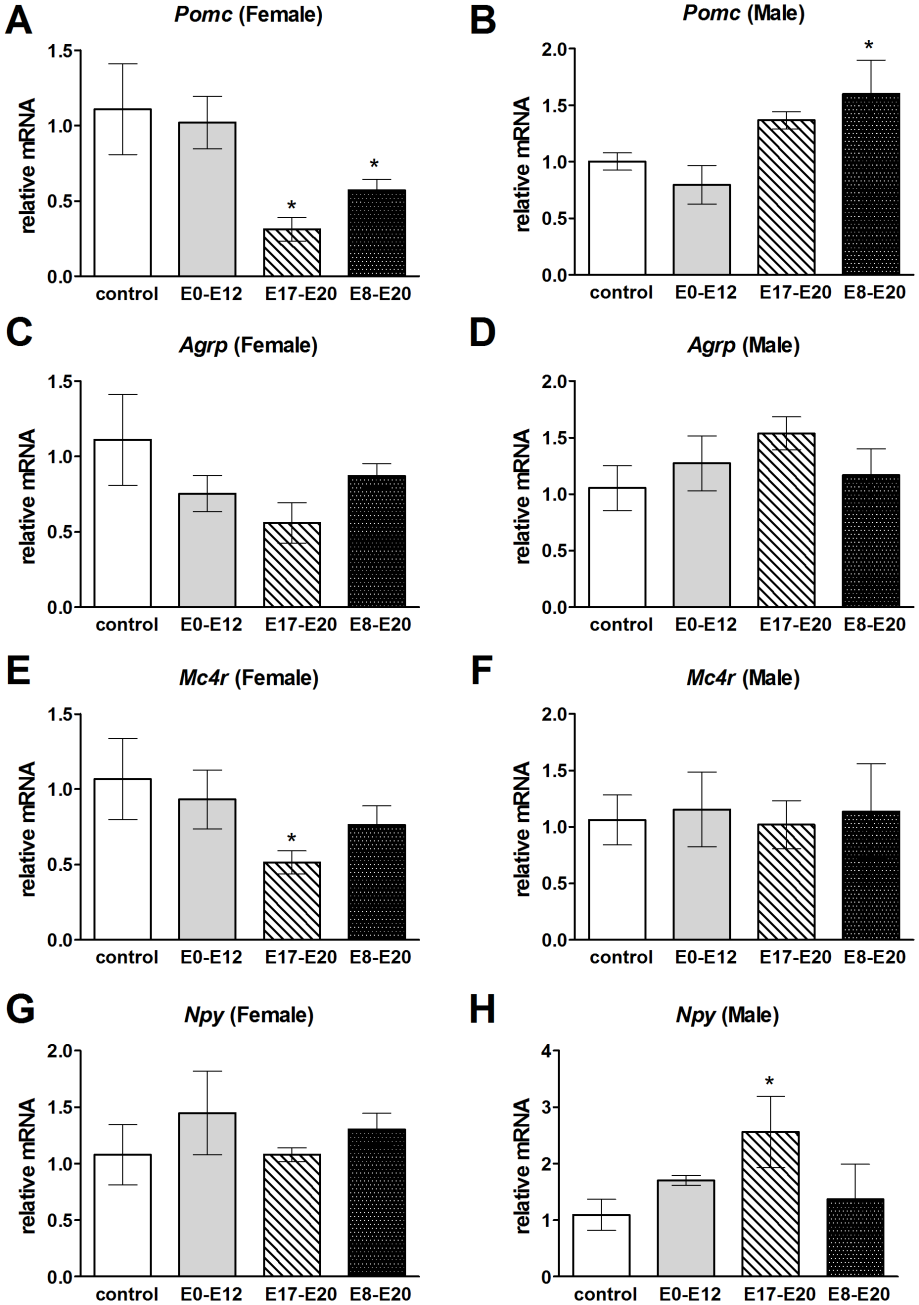
Sexually dimorphic hypothalamic gene expression patterns after TrkB inhibition during key embryonic time periods. qPCR measured hypothalamic gene expression changes of *Pomc* in A) female and B) male mice, *Agrp* in C) female and D) male mice, *Mc4r* in E) female and F) male mice, and *Npy* in G) female and H) male mice. All measurements were performed at four months of age on control mice or after 1NMPP1 treatment at E0–E12, E8–20 or E17–20. Data shown are mean ± SEM for each group. *Significant difference from all other groups, *P*<0.05.

We verified ISH results with qPCR to confirm that hypothalamic *Pomc* mRNA levels decreased in female mice at E17–E20 and E8–E20 relative to controls (Figure 7A) (E17–E20, n = 6; control, n = 4, *F*_1,10_ = 11.92, *P* = 0.01; E8–E20, n = 7; control, n = 4, *F*_1,11_ = 6.07, *P* = 0.03). This effect was not observed in male mice. Instead, male mice treated with 1NMPP1 during E8–E20 demonstrated increased *Pomc* expression relative to controls (E8–E20, n = 4; control, n = 5, *F*_1,9_ = 12.17, *P* = 0.005), and no change was observed for male mice treated at E17–E20 (E17–E20, n = 4; control, n = 5, *F*_1,9_ = 3.92, *P* = 0.07) (Figure 7B). Both female and male mice showed no change in hypothalamic *Agrp* mRNA levels following TrkB inhibition at any developmental stage (Figure 7C and D, respectively). Interestingly, female mice demonstrated decreased *Mc4r* expression at E17–E20 (E17–E20, n = 6; control, n = 4, *F*_1,10_ = 6.44, *P* = 0.03), but not at E8–E20 (E8–E20, n = 7; control, n = 4, *F*_1,11_ = 1.17, *P* = 0.30), relative to controls (Figure 7E). This suggests that female mice with TrkB inhibition from E17–E20 may have overall decreased melanocortin signaling since both *Pomc* and *Mc4r* mRNA levels decreased. Unlike in female mice, TrkB inhibition in male mice during embryonic development did not decrease *Mc4r* expression (Figure 7F). Female mice in which TrkB was inhibited during embryonic development did not have altered hypothalamic *Npy* mRNA levels (Figure 7G); in contrast, TrkB inhibition in male mice at E17–E20 resulted in increased *Npy* expression (Figure 7H) (E17–E20, n = 4; control, n = 5, *F*_1,9_ = 14.17, *P* = 0.003).

## Discussion

The formation of neural circuits in the hypothalamus during embryonic and early postnatal development is shaped by many of the same factors (e.g., leptin, estrogen, testosterone) [1], [2], [5], [6]. However, the role of Bdnf/TrkB signaling during the formation of these hypothalamic neural circuits has not been previously investigated. We show that chemical inhibition of TrkB during the period in which hypothalamic circuitry development results in obesity and sexually dimorphic patterns of gene expression that continue into adulthood. Specifically, inhibition of TrkB when connections between the VMH and PVN are being formed [40] resulted in sexually dimorphic changes in hypothalamic expression of genes involved in the control of energy balance. Further, we also observed that short-term inhibition of TrkB signaling in adults leads to long-lasting and irreversible weight gain. We identified key developmental time windows in which TrkB inhibition contributes to long-term obesity of both male and female mice, which stems from sexually dimorphic neuropeptide expression in the hypothalamus of genes known to regulate whole-body energy balance.

Many key physiological processes are regulated by the VMH, including glucose homeostasis and appetite control [44]–[47]. The developmental pattern of neuronal projections to the VMH is unique because it occurs during the prenatal period, unlike many other nuclei of the hypothalamus known to regulate whole-body energy balance [40]. Efferent VMH projections have been analyzed at E17.5, revealing the presence of two separate ascending pathways traveling to the medial basal forebrain and three descending projections traveling to the caudal portion of the brain [40]. One of the two ascending tracts branch off to form the VMH projections to the PVN. Interestingly, inhibition of TrkB (E17–E20) resulted in both postnatal and adult obesity and sex-specific changes in hypothalamic gene expression corresponding to the period in which VMH projections to the PVN are first formed.

Mice deficient in TrkB do not show alterations in sexual development, whereas mice deficient in steroidogenic factor 1 (*Sf1, NR5A1*) exhibit gross alterations in sexual development. Male mice lacking *Sf1* develop female genitalia [48]–[50]. Interestingly, *Sf1* is a molecular marker for the VMH and is known to regulate development of both VMH neural projections and the cytoarchitecture of the nuclei [25], [48], [51]. *Sf1* knockout mice have decreased *Bdnf* expression in the VMH by E17.5, suggesting that downregulation of the Bdnf signaling pathway modulated by *Sf1* could regulate sexually dimorphic gene expression patterns [25]. This observation implies that the phenotype seen following TrkB inhibition during E17–E20 may result from disrupted TrkB signaling in a hypothalamic region known to regulate sexually dimorphic behavior, such as the VMH. Given that neural projections from the VMH are forming during this period, we propose that the Bdnf/TrkB pathway may modulate the development of efferent VMH projections and/or survival of VMH neurons during E17–E20 and that inhibition of this pathway results in adult obesity.

Leptin levels positively correlate with fat mass; leptin decreases *Agrp* and increases *Pomc* expression in the hypothalamus [43], [52], whereas *Npy* expression contributes to leptin resistance [53]–[55]. *Bdnf* heterozygote mice have increased leptin levels and no change in *LepR* expression, suggesting that Bdnf functions downstream or independently of the LepR/Npy pathway [13]. *LepR* expression was not altered in mice carrying a truncated form of the long 3′UTR of *Bdnf*, despite leptin resistance in these mice [56]. Similarly, we show that acute inhibition of TrkB signaling alters neither *LepR* (long form) expression (control males, 1.05± 0.19; control females,1.17±0.42; E8–E20 males, 1.12±0.19; E8–E20 females, 1.98±0.82; E17–E20 males, 1.92±0.71; E17–E20 females, 0.74±0.13) nor *Agrp* expression. These data suggest that acute developmental disruption of TrkB signaling may alter hypothalamic neural circuits involved in leptin signaling (e.g. *Npy* or *Pomc/Mc4r* expression), although this needs to be investigated further.

TrkB inhibition at E8–E20 increased fat mass, body weight, and hypothalamic *Pomc* mRNA levels in male mice. However, TrkB inhibition at E17–E20 increased fat mass and body weight and decreased hypothalamic melanocortinergic tone in female mice. Increased Pomc may alter components of food intake (e.g., satiety signals), which would result in small changes in meal patterns that accumulate to long-term changes in body weight gain over time. Thus, we propose that TrkB inhibition during embryonic development (E8–E20) in male mice, but not female mice, may trigger an overall phenotype that resembles mice carrying a targeted deletion of *Bdnf* or *TrkB*. Male mice treated at E17–E20 increased hypothalamic *Npy* levels, again suggesting that TrkB inhibition in male mice may alter signals associated with meal patterns, with only a trend toward increased fat mass and no change in body weight. Here, we present acute inhibition of TrkB signaling during specific developmental stages and demonstrate that the body weight and body composition phenotype, as well as the hypothalamic neuropeptide profile for *LepR* and *Npy*, resemble that of *Bdnf* heterozygote mice [13] or *TrkB* hypomorphs [14]. These phenotypes persist even after TrkB signaling is restored [20]. Chronic delivery of Bdnf restores the body weight phenotype of *Bdnf*-deficient mice [13], [56]; therefore, it remains to be determined whether overexpressing Bdnf (e.g. AAV delivery or chronic protein delivery), after acute TrkB inhibition and reactivation, could restore the body weight phenotype.

*Bdnf* heterozygote and *TrkB* hypomorph mice are obese [13], [14], suggesting that treatment of *TrkB^F616A/+^* mice will produce a similar phenotype as observed following treatment of *TrkB^F616A/F616A^* animals. The maternal influences of the offspring treated with 1NMPP1 for 4, 7, or even 14 days may indirectly influence the developing embryos/pups during or after this time. However, many confounding studies that analyze mutant offspring from mutant mothers, including the obese *Bdnf* heterozygote or the *TrkB* hypomorph, demonstrate long-term physiological changes and exhibit a potential confound in all studies. In order to differentiate maternal versus embryonic contributions resulting from stage-specific inhibition of TrkB signaling, mating *TrkB^F616A/+^* mice to generate all genotypes in the offspring would be beneficial. For example, the offspring generated from mating *TrkB^F616A/+^* combined with stage-specific embryonic inhibition of TrkB, may generate similar phenotypes in all the offspring, suggesting that maternal influences are dominant over embryonic influences. Here, the temporally-restricted and reversible inhibition of TrkB signaling makes it possible to determine precisely when Bdnf acts to modulate body weight. It remains to be determined whether TrkB inhibition alters maternal feeding behavior to induce sex-specific changes in hypothalamic neural circuitry of offspring. However, the E14–E18 and E0–E12 groups treated with 1NMPP1 exhibited very modest and short-term alterations in body weight (e.g., increased and decreased body weight, respectively), suggesting a modest contribution of maternal influence on the observed phenotype.

TrkB inhibition in adult animals also led to a long-lasting and persistent increase in body weight. Long-term overfeeding both early in life and in adulthood can persistently increase baseline body weight, which can be difficult to reduce by food restriction alone. These alterations may result from structural changes in homeostatic regulatory circuitry within the hypothalamus [57]. Structural plasticity may be mediated by changes in synaptic connectivity between existing hypothalamic neurons or the integration of newborn neurons into existing circuitry. Indeed, intracerebroventricular (ICV) delivery of Bdnf increases neurogenesis in the hypothalamic parenchyma of adult rats [58], but not the subventricular zone [59], suggesting that neurogenesis may be one possible mechanism by which inhibition of TrkB signaling might lead to long-term changes in body weight. Bdnf also binds p75 neurotrophin receptor (*p75^Trk^*), which modulates neurogenesis in the embryo and in the adult olfactory bulb [60], [61]. This raises the possibility that adult hypothalamic neurogenesis may involve the action of both p75^Trk^ and TrkB. However, P75^Trk^ is activated by multiple neurotrophic factors (e.g. Nerve Growth Factor, Neurotrophin-3, Neurotrophin-4/5) and has been shown to activate cell death [62]. TrkB inhibition may increase Bdnf/p75^Trk^ signaling in order to shift the balance between neurogenesis or cell death [58], [60]–[62], which has not been thoroughly investigated in the hypothalamus. It remains to be determined whether changes in neurogenesis or cell death result from stage-specific inhibition of TrkB and how this interacts with p75^Trk^ signaling.

Neurogenesis in the adult hypothalamus is also induced by ICV delivery of ciliary neurotrophic factor (Cntf). Cntf also reduces body weight, although both effects are reversed by cytosine-β-D-arabinofuranoside (AraC) [63], [64]. High fat diet-induced obesity (DIO) also broadly inhibits hypothalamic neurogenesis in adult male mice [65], [66]. In contrast, DIO stimulates neurogenesis in the median eminence of both neonatal and adult female mice, leading to a long-term increase in body weight [67]. These data collectively imply that dietary-induced changes in hypothalamic neurogenesis may play a central role in reshaping homeostatic neural circuitry that regulates body weight. Bdnf signaling may regulate the proliferation, differentiation, or survival of these newly generated neurons. The potential sex-specific differences reported in the regulation of adult neurogenesis by DIO [65]–[67] may likewise partially underlie the greater increase in body weight of adult female mice following TrkB inhibition.

Our studies demonstrate that Bdnf/TrkB signaling modulates body weight, body composition, and hypothalamic gene expression at specific time points throughout the lifespan of the mouse. Further, TrkB inhibition has differential stage and sex-specific effects. Over the last 20 years, the incidence of obesity in infants and children under the age of five years has greatly increased [68]–[70]. This raises concern because obesity during infancy and childhood increases susceptibility to adult diseases [71]. Given the increasing trend in infant and childhood obesity, determining the critical time frame during pre- and postnatal development for which TrkB signaling influences obesity has important implications. Interestingly, high-fat/high-sugar diets decrease hypothalamic expression of both *Bdnf* and *TrkB*, suggesting that diet composition (e.g. macronutrients) may influence this pathway [8]–[11]. Our results show that downregulation of the Bdnf/TrkB signaling pathway during critical developmental time periods leads to a dramatic short-term increase in body weight. Although TrkB signaling has been reactivated and body weight modestly decreases over time, it never returns to baseline in adulthood. This suggests that the homeostatic set point for maintaining body weight has been permanently reset. Finally and unexpectedly, adult animals also show rapid and stable increased body weight after acute inhibition of TrkB signaling, even after TrkB reactivation. Adults do not lose weight gained following 1NMPP1 treatment, in sharp contrast to what is seen following late embryonic and neonatal treatment. This indicates a fundamentally different requirement of Bdnf signaling in regulation of body weight in adult animals, possibly through regulation of hypothalamic neurogenesis.

### Conclusions

Using chemical-genetics, we have systematically characterized the effects of endogenous TrkB inhibition at different developmental stages on the regulation of body weight and adult obesity (Table 1). We observed a continual requirement for TrkB signaling in regulating body weight throughout the mouse lifespan. We further demonstrated an unexpected sexually dimorphic effect of TrkB inhibition on both body weight and hypothalamic expression of genes known to regulate food intake and body weight (Table 1). Because hypothalamic TrkB signaling can also be modulated by dietary cues [8], [10], [11], this work has important potential implications for human health.

**Table 1:** TrkB inhibition alters body composition, body weight, and neuropeptide levels in a stage- and sex-specific manner.

*1NMPP1 Treatment*	*Body Weight*	*Body Composition*		*Neuropeptide*
E17–E20	**M - NC**	**Fat mass (M) - #↑**	**M**	**Pomc – NC**
	F - ↑	Fat mass (F)- ↑	F	Pomc – ↓
		**Fat-free mass (M) - NC**	**M**	**Agrp – NC**
		Fat-free mass (F)- ↑	F	Agrp – NC
			**M**	**Mc4r – NC**
			F	Mc4r – ↓
			**M**	**Npy – ↑**
			F	Npy – NC
E8–E20	**M - ↑**	**Fat mass (M) - ↑**	**M**	**Pomc – ↑**
	F - NC	Fat mass (F) - NC	F	Pomc – ↓
		**Fat-free mass (M) - NC**	**M**	**Agrp – NC**
		Fat-free mass (F) - ↑	F	Agrp – NC
			**M**	**Mc4r – NC**
			F	Mc4r – NC
			**M**	**Npy – NC**
			F	Npy – NC

Sex-specific changes in body weight, body composition and hypothalamic neuropeptide expression after embryonic TrkB inhibition. Male (M) data presented in bold font and female (F) data is presented in standard font. ↑- increase, ↓- decrease, and NC - no change, ^#^represents P = 0.06.
